# *Mycobacterium bovis-*BCG Vaccination Induces Specific Pulmonary Transcriptome Biosignatures in Mice

**DOI:** 10.1371/journal.pone.0011319

**Published:** 2010-06-28

**Authors:** Elihu Aranday Cortes, Daryan Kaveh, Javier Nunez-Garcia, Philip J. Hogarth, H. Martin Vordermeier

**Affiliations:** TB Research Group, Veterinary Laboratories Agency, Addlestone, United Kingdom; Universita di Sassari, Italy

## Abstract

**Background:**

In the present study, we applied microarray technology to define biosignatures by microarray transcriptome analysis in lung and spleen samples after BCG vaccination and *M. bovis* infection of BALB/c mice. The aims were two-fold, namely to define biosignatures that could predict vaccine success before challenge, and biomarker patterns that correlated with anamnestic protective responses following exposure to virulent *M. bovis.* Further, these biosignatures should be detectable without *in vitro* antigenic challenge.

**Principal Findings:**

After BCG vaccination, we defined a specific pulmonary gene expression signature related to the connective tissue development and function network that predicted vaccine success before *M. bovis* challenge. In addition, a Th17-related cytokine profile was found that correlated with vaccine-induced protective immunity following infection with virulent *M. bovis* in the lung as well as additional genes that were up-regulated in the spleens of vaccinated animals post-infection related to neutrophil biology and inflammation.

**Conclusions:**

This study has therefore prioritized both biomarkers predicting vaccination success before challenge and bio-signatures that are potentially associated with protective immune responses that will be useful to evaluate future vaccine candidates.

## Introduction

BCG is the most widely used human vaccine with an excellent and unmatched safety record in immuno-competent humans. Protection against TB conferred by BCG is thought to be mediated by the induction of cell mediated immune responses characterized by the cytokine IFN-γ. Although necessary, IFN-γ alone is not sufficient for protection and therefore, it is unreliable as a predictor or correlate of protection (rev. in [1]). More reliable markers of protection would accelerate the development of novel and more effective vaccination strategies against human and bovine tuberculosis.

*Biomics* approaches (transcripto- proteo- and metabolo- mics) have changed the way researchers are conducting experiments. For example, transcriptome analysis measure changes in global gene expression comparing different gene expression profiles between different experimental conditions. This approach has been used, for example, in cancer related studies in order to obtain prognostic values [2] and predicting therapeutic outcome [3]. In addition, these approaches are now also being used to identify biomarkers of infection [4] or to study vaccine-induced responses, such as after yellow fever vaccination [5].

The effect of BCG vaccination to protect cattle against *M. bovis* infection has been studied since 1911, and several recent reviews have covered these studies in details [1], [6]. The results of the majority of experimental vaccine challenge studies have demonstrated a considerable degree of protection. Further, BCG vaccination constitutes a good model vaccine to investigate the mechanisms of protective immunity against tuberculosis infection in small and large animal models. We have shown that the murine *M. bovis* infection model is predictive of BCG and sub-unit vaccination success in cattle [7], and have therefore applied microarray technology to define biosignatures from the whole transcriptome in lung and spleens of BALB/c mice following BCG vaccination and *M. bovis* infection. We identified specific pulmonary gene expression signatures related to connective tissue development and a Th17-related cytokine profile. These signatures predicted vaccine success prior to challenge, or correlated with vaccine-induced protective immunity following infection with virulent *M. bovis*.

## Materials and Methods

### Animals

Female BALB/C mice were obtained from SPF facilities at Charles River Laboratories, Margate, UK. All animals were housed in appropriate biological containment facilities at VLA and work was carried out in accordance with the UK Home Office Animal (Scientific Procedures) Act 1986, following approval by the VLA Ethical Review Board and the UK Home Office.

### Mycobacteria

The vaccination strain used was *M. bovis* BCG Danish strain 1331 (SSI, Copenhagen, Denmark) which was reconstituted from freeze-dried stocks stored at 4°C in Sauton's medium supplied as per instructions. *M. bovis* isolate AF2122/97 grown to mid log phase in Middlebrook 7H9 broth supplemented with 4.16 g/L pyruvic acid, 10% (v/v) oleic acid, albumin, dextrose, and catalase (OADC) and 0.05% (v/v) Tween 80, subsequently stored at −80°C, was used for all virulent challenges.

### Immunisation and mycobacterial challenge

Two groups of 15 mice each were immunised by a single intradermal injection of 2×10^5^ CFU of *M. bovis* BCG (Vaccinated), or Sauton's medium (Unvaccinated). Vaccinations were carried out into the base of the tail in 50 µl volumes. Six weeks later 5 mice from each group were euthanized for immunological analyses and the remaining mice from each group were challenged with approx 600 CFU *M. bovis* via the intranasal route [8]. At days 3 and 14 post challenge five mice per group were euthanized and spleens and lungs harvested (Table 1). To validate results obtained from samples before infection, we also prepared cells (as described below) from a further experiment conducted in the same way as described above. In this experiment, 5 vaccinated and 5 unvaccinated control mice were euthanized 6 weeks post-vaccination and cells and RNA prepared for qRT-PCR analysis as described below.

**Table 1:** Vaccination and challenge design.

	Pre-Infection	Post-Infection	
		*Day 3*	*Day 14*
**Naïve**	5 animals (UU)	5 animals (UI3d)	5 animals (UI14d)
**BCG Vaccinated**	5 animals (VU)	5 animals (VI3d)	5 animals (VI14d)

Experimental design used for microarray study of gene expression in spleen and lung from vaccinated and unvaccinated mice. 6 groups with 5 animals per group and one microarray for each animal (30 microarrays) were used. Definition of abbreviations used: UU =  Unvaccinated prior to infection; UI3d, UI14d =  Unvaccinated euthanized 3 or 14 days post-*M. bovis* infection; VU =  BCG Vaccinated Prior infection; VI3d, VI14d =  Vaccinated 3 or 14 days post-infection.

### Cell isolation

Five mice per group were euthanized to evaluate the immune response of splenic lymphocytes. Spleen cells were prepared by passage through a 40 µm cell strainer into DMEM supplemented with 10% (v/v) foetal calf serum (FCS) and antibiotics (100 U/ml penicillin and 100 µg/ml streptomycin) (Gibco, UK) Following washing at 300× g for 10 minutes cells were suspended at 5×10^6^/ml for assay.

Lung cells were isolated as follows. Briefly, thoracic cavities were opened, descending aorta transected and sterile HBSS gently injected into right heart ventricles to perfuse lungs. Lungs were excised, resuspended into digestion media (DMEM supplemented plus 10 U/ml DNAse II (Sigma) and 150 U/ml collagenase type I (Gibco)) and incubated for 1 hour at 37°C with gentle agitation (200 rpm). After digestion lungs were prepared by passage through 100 µm cell strainer into supplemented DMEM. Following washing at 1600 rpm for 5 minutes, cells were poured into another tube through a 40 µm cell strainer, washed and used at 5×10^5^/well.

### Determination of bacterial load

Spleen and lungs were removed and homogenised in distilled H_2_O containing 0.05% v/v Tween 80 using a 10 mm coarse dispersing homogeniser. Serial dilutions were plated onto modified Middlebrook 7H11 agar media (Gallagher, et. al. 1977) and incubated at 37°C for 4 weeks prior to Colony Forming Units (CFU) determination.

### RNA preparation and microarray hybridization

Spleen and lung cells prepared as and identical to the samples described above were pelleted, and then resuspended in Trizol and stored at −80°C until further processing. Trizol was chosen as storage medium because our validation experiments had demonstrated that *M. bovis* was effectively killed in Trizol. Therefore, further processing could be performed outside a biosafety containment level 3 (CL3) facility. RNA was isolated from spleen and lung cells derived from BCG vaccinated and control mice before and after *M. bovis* challenge using standard RNA extraction protocols (Miltenyi Biotech Ltd, Bergisch Gladbach, Germany). The quality of RNA samples was assessed using the Agilent 2100 Bioanalyzer platform. All RNA samples revealed acceptable RNA Integrity Number (RIN) values between 6.8 and 9.5. For the linear T7-based amplification step, 0.5 µg of each total RNA samples was used. To produce Cy3-labeled cDNA, the RNA samples were amplified and labeled using the Agilent Low RNA Input Linear Amp Kit following the manufacturer's protocol. Yields of cDNA and the dye-incorporation rate were measured with the ND-1000 Spectrophotometer. The hybridization procedure was performed according to the Agilent 60-mer oligo microarray processing protocol using the Agilent Gene Expression Hybridization Kit. Briefly, 1.65 µg Cy3-labeled fragmented cDNA in hybridization buffer was hybridized overnight (17 hours, 65°C) to Agilent Whole Mouse Genome Oligo Microarrays 4×44K using Agilent's recommended hybridization chamber and oven. Finally, the microarrays were washed once with 6× SSPE buffer (3.6 M NaCl, 0.2 M NaH_2_PO_4_, 0.02 M EDTA pH 7.4) containing 0.005% N-lauroylsarcosine for 1 min at room termperature followed by a second wash with preheated 0.06× SSPE buffer (37°C) containing 0.005% N-lauroylsarcosine for 1 min. The last washing step was performed with acetonitrile for 30 seconds.

### Normalization, filtering procedures and data analysis

Fluorescence signals of the hybridized Agilent Microarrays were detected using Agilent's Microarray Scanner System. The Agilent Feature Software (FES) was used to read out and process the microarray image files. The software determines feature intensities (including background subtraction), rejects outliers and calculates statistical confidences. For determination of differential gene expression FES derived output data files were further analyzed using GeneSpring GX 11 (Agilent). The default normalization for Agilent one-color data in the GeneSpring GX program is quantile normalization following Agilent' guidelines. This was done across all arrays and caused all distributions to be the same. Therefore, this non-linear normalization corrects array biases. After normalization and baseline transformation performed using median of UU samples as a baseline; we decided to focus on those genes that reliably change their expression, then we filtered the microarrays following three conditions: 1) *Filter by value.* Genes that do not have normalized signal intensity values of more than −0.5 and 0.5 were disregarded. 2) *Filter by flags.* All the genes with flag present in at least 100% of the values in any 1 out of the 6 conditions (see Table 1) were considered. Flags are attributes that denote the quality of the entities. Using these attribute values in GeneSpring we filtered the genes. based on the feature quality on the chip, like signal saturation and signal uniformity. The genes which are given a low significance attribute in the data file were marked as 'Absent' and those with high significance values were marked as ‘Present’. Thus, the following flags are possible: ‘present’ (transcription), ‘marginal’ (could be transcription, linking or mismatch) or absent (no transcription). 3) Filter by percentile. All the genes with normalized signal intensity values between 25 and 100 in any 1 out of the 6 conditions (see Table 1) were also considered. Finally, all the genes in coincidence between filtering by flags group and filtering by percentile group were kept for statistical analysis.

After filtering, analysis of variance was applied to compare mean expression levels in each analysis. Data were considered significant when the Benjamini Hochberg false discovery rate (FDR) for the comparison under analysis was <0.05, and the significance level was <0.05. In order to focus on highly regulated genes, we also restricted the majority of the analysis to genes with changes in expression levels of at least 1.7-fold change in all the conditions.

Lists of genes resulting from analyses were submitted to Ingenuity Pathway Analysis (IPA; Ingenuity® Systems, Redwood City, Ca, www.ingenuity.com) in order to obtain the most significant networks and canonical pathways related. By default p<0.05 was used in all the analyses. All data set can be downloaded from Gene Expression Omnibus public data base at www.ncbi.nml.nih.gov/geo/ with the GEO accession number GSE21149.

### Real-time quantitative PCR validation

Fast SYBR Green real time PCR was used to validate microarray gene expression results. cDNA was synthesized from total RNA samples using reverse transcription with Verso reverse transcriptase following the manufacturers protocol (Thermo Scientific, Epson, Surrey, UK). QRT-PCR analysis was performed using the Applied Biosystem ABI 7500 Fast machine in triplicate from five biological independent samples of total RNA from the lung and spleen of unvaccinated and vaccinated animals. The fold increase in signal over the G3PDH housekeeping gene was determined using the 2^−ΔΔct^ calculation [9].

## Results

### Protective efficacy

Groups of 5 BALB/c mice, vaccinated with *M. bovis* BCG or sham vaccinated, were challenged with *M. bovis* via the intranasal route. Bacterial burdens (Table 2) were determined 28 days post infection. Host immune and transcriptional responses were determined prior to, and 3 and 14 days after infection. The data shown in table 2 demonstrated that BCG vaccination induced significant protection in spleens and lungs of vaccinated mice.

**Table 2:** *M. bovis* bacterial loads in the spleen and lung.

	Spleen (Log_10_ cfu)	S.E.	Spleen Log_10_ protection	Lung (Log_10_ cfu)	S.E.	Lung Log_10_ protection
Controls (n = 5)	4.86	0.17	-	6.95	0.30	-
BCG (n = 5)	3.38	0.10	1.48 ***	5.07	0.14	1.88 **

**p<0.005 *** p<0.0005 vs. control (two-tailed t test with Welch correction).

### BCG vaccination induces a specific pulmonary gene-expression signature

**Figure 1:**
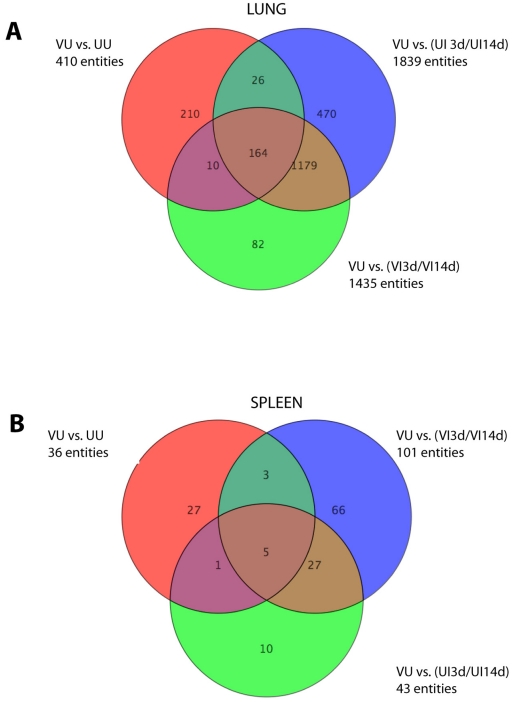
Venn diagram showing the distribution of genes that define vaccine success after BCG vaccination but before *M. bovis* challenge in lung and spleen. Comparisons of at least 1.7-times over- or under-expressed gene obtained for UU, VI and UI samples relative to VU, resulting in gene expression patterns that predict protection in lung (A) and in spleen (B). A list of these genes is provided in Table S1.

**Figure 2:**
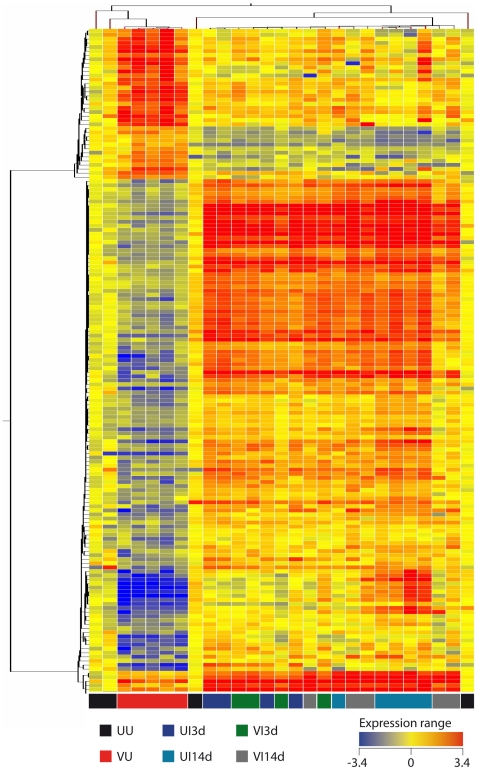
Pulmonary gene signature after BCG vaccination but before *M. bovis* challenge. By using unsupervised hierarchical clustering of transcripts and samples with centroid linkage and Pearson centered measure, lung samples were segregated into two distinct groups based on the 164 genes identified previously (Fig.1A). All VU samples (red squares at the bottom) were clustered together with two UU samples. Bright red represents a 3.4-fold increase in expression above the mean and bright blue represents a 3.4-fold change decrease in the expression from the average to all UU mice. Yellow indicates the unchanged expression level of a partiuclar gene between vaccinated and uninfected control mice (VU and UU groups, respectively).

In order to define a specific biosignature that can identify vaccine success after BCG vaccination but before *M. bovis* challenge (predictors of protection), lung and spleen samples were collected and RNA prepared without prior *in vitro* stimulation (*ex vivo* samples). We compared samples from BCG vaccinated animals (VU samples, see Table 1 for definitions of groups) against all the other groups and time points post-vaccination and post-challenge (UI3d/UI14d and VI3d/VI14d). Genes were considered significant when their corrected ρ-values were below 0.05 with more than 1.7 fold changes of expression. Applying these criteria, the expression of 169 (164 genes in the lungs, Figure 1A, and 5 genes in the spleens, Figure 1B) genes was significantly modulated in the BCG vaccinated VU group compared to unvaccinated groups before and after challenge, as well as vaccinated groups after challenge (Fig. 1). Unsupervised hierarchical cluster was performed using a centroid linkage with a Person centered measure. VU samples were found to cluster together with a clear differentiation between VU and either VI or UI samples. Figure 2 shows the analysis of the lung samples which demonstrated a specific gene expression profile after vaccination with BCG six weeks after vaccination (VU), with 121 genes down-regulated and 43 up-regulated compared with the gene expression in all other groups and time points (UU, VI3d/14d and UI3d/14d, Fig. 2 and Table S1).

### Ingenuity Pathway Analysis

Ingenuity Pathway Analysis (IPA) was performed on these 164 genes up-or-down-regulated in the lung of vaccinated animal and revealed that they were highly significantly related with connective tissue development and function (32 genes, ρ-value = 0.65E-04), cell death (26 genes, ρ-value = 3.93E-05), cellular development (22 genes, p-value = 2.15E-04), cell-to-cell signaling and interaction (n = 19 genes, p-value = 4.92E-05) and cellular assembly and organization (9 genes, p-value = 4.90E-05). In contrast, the five genes significantly over or under-expressed in spleen samples from vaccinated mice were all poorly annotated at the time of the analysis (classified as hypothetical or unclassifiable genes).

**Figure 3:**
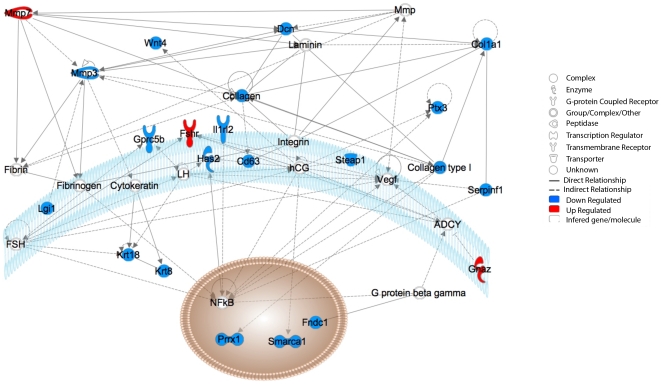
Connective tissue disorder network genes are differentially modulated after BCG vaccination but before *M. bovis* challenge in lungs. The connective tissue disorder network was identified after Ingenuity Analysis (p-value 1.65E-4) based on a subset of 164 genes identified previously in lung (Fig.1A). The relationship between individual genes is depicted in this figure. Red: up-regulated; Blue: down-regulated; White: not found to be modulated.

Most of the genes involved directly or indirectly related with connective tissue development and function network were down-regulated after vaccination alone but were consistently up-regulated after challenged with *M. bovis* (Figures 2 and Table S1). For example, the gene encoding matrix metalloproteinase-3 (mmp3) was down-regulated 36-fold in VU samples compared with VI and UI samples and down-regulated 2.5-fold compared with UU samples (Table S1). In contrast, another gene from the same MMP family, mmp7 was up-regulated only in VU samples with a 5-fold change compared with all the other samples. Other genes related to this pathway that were modulated differentially in VU lungs were decorin and pentraxin 3 (Figure 3 and Table S1).

**Figure 4:**
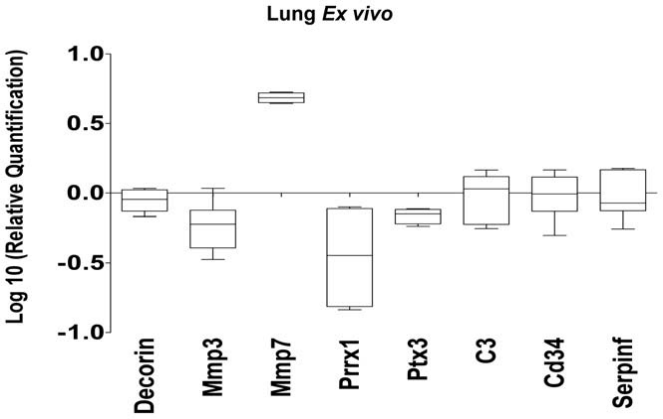
Confirmation of the modulation of selected genes by quantitative RT-PCR. To confirm the expression patterns of some genes identified in the microarray studies, real-time RT-PCR (QRT-PCR) was performed using total RNA collected from vaccinated and unvaccinated mice prior to infection form an experiment independent from the original experiment that provided samples for the microarray analysis. Data are expressed as log10 relative expression levels in vaccinated compared to naïve control mice.

The validity of these micro-array data was confirmed by conventional qRT-PCR analysis of a selection of genes including mmp3, mmp7, decorin and pentraxin 3 (figure 4) using lung samples from VU and UU mice obtained from an independent experiment using BCG vaccinated and control mice vaccinated identically to the mice from the experiment used for the array analysis.

### Correlates of protection

**Figure 5:**
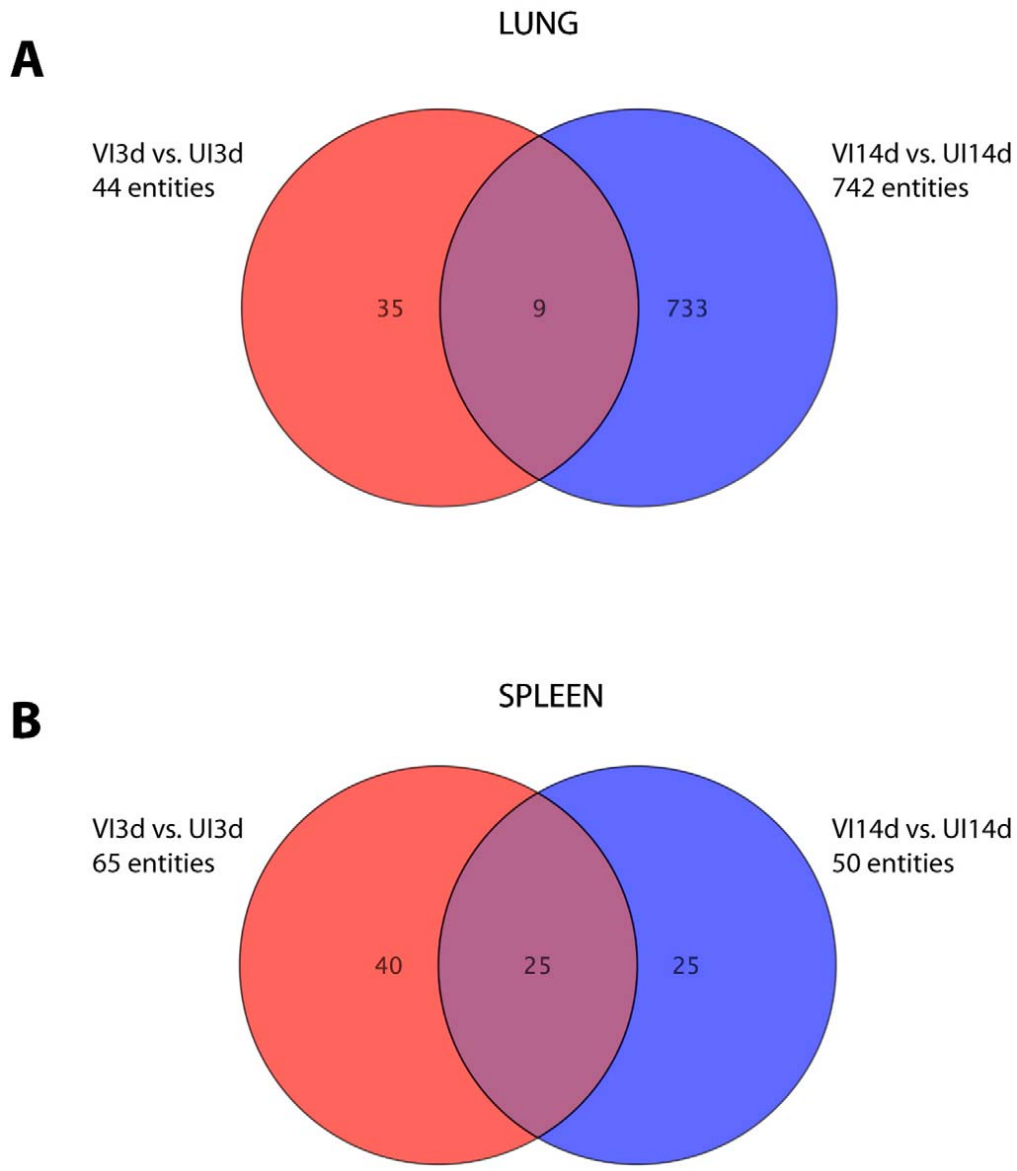
Venn diagrams of genes defining protective host responses after *M. bovis* challenge. Comparisons of genes that were over or under-expressed at least 1.7- times in the lungs and spleens of vaccinated compared to unvaccinated mice after *M. bovis* challenge are shown (UI samples relative to VI samples at day 3 and 14 post challenge). (A) Lung cell responses; (B) spleen cells responses (B). Genes involved are listed in Table S2).

**Figure 6:**
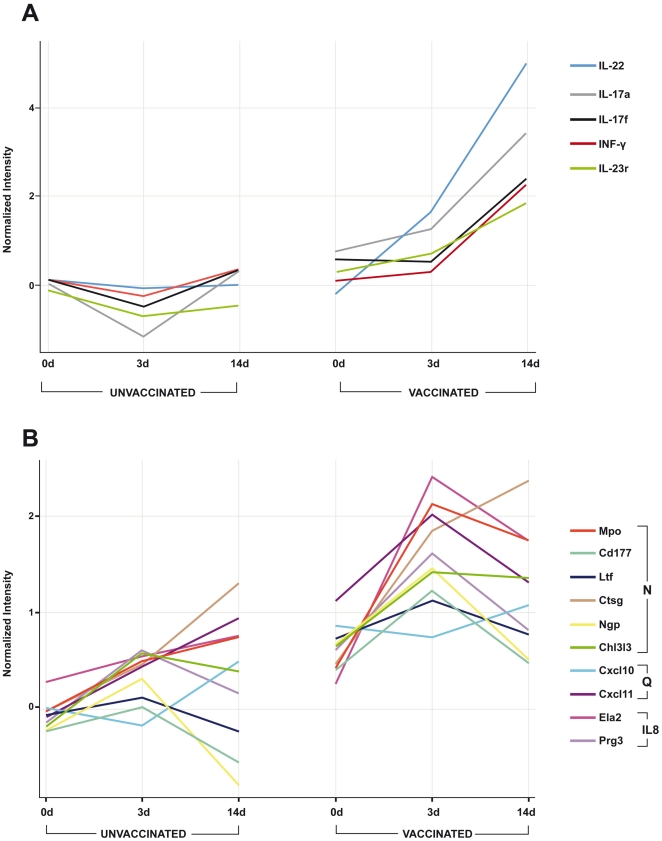
Transcription profiles in lung and spleen cells of selected genes differentially modulated between vaccinated and unvaccinated mice post-infection. Each line shows expression levels determined by microarray analysis in lung (A) and spleen cells (B) of individual transcripts between unvaccinated and vaccinated samples. (A) Pulmonary (lung cell) expression profiles of TH17 pathway related genes. (B) Spleen cell expression profiles of genes related to neutrophil-biology. For (B): N = genes related to neutrophil activity; Q =  genes related to neutrophil chemotaxis; IL8 =  genes related to interleukin-8 biosynthesis.

In order to define biomarker signatures indicative of a protective host response in BCG vaccinated animals following *M. bovis* infection, i.e. potential correlates of protection, the differences in the transcriptional profiles between unvaccinated mice 3 and 14 days after infection (UI3d and UI14d) were compared against vaccinated mice at the same time points (VI3d, VI14d). At day 3 and 14-post infection, 44 and 742 genes, respectively, were significantly differentially expressed between vaccinated and unvaccinated mice in lung samples, respectively. Only 9 genes of these 786 genes were differentially expressed at both time-points post-infection (Fig. 5A). When we assessed the functions of these 786 differentially expressed genes, we found that genes associated with the TH17 differentiation and effector pathways were prominent amongst them. For example, both expression of Interleukin-17A and F encoding genes was up- regulated in the lung samples of BCG vaccinated animals both 3 and 14 days after challenge with *M. bovis*, whilst expression of the genes encoding interleukin-22 and interleukin-23 receptor were up-regulated in lung samples from vaccinated animals 14 days after challenge with *M. bovis* (Figure 6A). As expected, IFN-γ was also strongly and significantly up-regulated in the lungs of protected mice (Fig. 6A).

After comparative analysis of the spleen samples we found 65 and 50 genes, respectively, to be differentially expressed at days 3 and 14 between vaccinated and unvaccinated animals (Fig. 6B), with 25 genes differentially expressed at both time points post-infection. For this set of genes IPA analysis revealed that genes associated with inflammatory responses were found to be the primary network up-regulated (score 39) in vaccinated samples. For example, three days post-challenge, the eosinophil related genes eosinophil peroxidase (Epx), proteoglycan 2 and eosinophil granule major basic protein (MBP2) were up-regulated (Table S2). Interestingly, 8/25 of these up-regulated genes listed in Table S2 were neutrophil-related (Figure 6B): myeloperosidase (mpo), CD177, neutrophilic granule protein (npg), lactotransferrin (ltf), cathepsin G (ctsg), chitinase 3-like 3 (chi3l3); chemokines related to neutrophil chemotaxis (Figure 6B: cxcl-10, cxcl-11); or related to the IL-8 activity of neutrophils (Figure 6B: elastase-2 (ela2), proteoglucan 3 (prg3). Further, interferon related genes, for example, 2′-5′ oligoadenylate synthetase-like2 (oasl2) and interferon-induced protein tetrapeptide repeats 3 (ifit3); Lymphocyte Antigen 6 complex (ly6a); and Ficolin B (fcnb), a member of the collectin family, an intracellular scanvenger that recognize pathogen-associated molecular pattern on microbial surfaces [10], were also up-regulated in vaccinated compared to unvaccinated mice after challenge (Table S2).

## Discussion

This study probed transcriptional responses in BCG vaccinated and naïve mice before and after *M. bovis* challenge. The aims were two-fold, namely to define biosignatures that could predict vaccine success before challenge, and biomarker patterns that correlated with anamnestic protective responses following exposure to virulent *M. bovis.* As our objective was to define markers which are detectable without further *in vitro* antigenic stimulation, we prepared RNA from lung and spleen cells directly after cell preparation (*ex vivo* sampling).

Our results are consistent with previous studies, demonstrating that after vaccination with BCG a biosignature, rather than a single marker, will be required to predict the subsequent outcome of infection in respect to protection status [11], [12], [13], [14]. Significantly, following vaccination, we found that the differentially expressed genes in the BCG vaccinated unchallenged animals compared to all other groups were involved in connective tissue development and MMP protein function. One can therefore hypothesize that parenteral BCG vaccination resulted in re-modeling of the lung tissue conferring resistance to later areogenic infection. As it has been described that systemic BCG vaccination of mice leads to the dissemination of BCG throughout the body including the lungs [15], [16], one could further hypothesize that this effect was induced by the presence of live BCG in the lungs of vaccinated mice. We detected this differential expression of these connective tissue associated genes in our study 6 weeks post-vaccination, and it will be of interest to determine how long the up-regulation of these genes is detectable following vaccination. Although we have not determined BCG persistence in this experiments, we have found in a subsequent and still on-going experiment that BCG can be cultured in the lungs of a proportion of vaccinated mice for up to 4 months post-vaccination (Kaveh and Hogarth, unpublished observation), thus supporting our hypothesis. However, future studies need to be conducted, using for example gene knock out mice, to determine whether this tissue re-modeling is needed for protection or is only correlated with the presence of BCG in the lung tissue.

The proteins of the MMP family have been described to play a dual role in the eradication of infections. Lymphocyte migration, tissue remodeling, granuloma formation, defensin and cytokine activation are required by the host in order to control and eradicate successfully pathogens. However, MMP's also are responsible for excessive tissue damage, pulmonary cavitation and increased blood brain barrier permeability that would help pathogen dissemination or persistence in the host [17]. It had been shown that MMP7, also known as matrilysin, is involved in inflammatory and healing process in the lung [18]. For instance, *mmp7^−/−^* mice developed an attenuated asthmatic phenotype and airway epithelial expression of MMP-7 was critical for development-asthma like disease [19]. Conversly, in obliterative bronchiolitis [20], matrilisyn showed a protective role in chronic lung injury, regulating a population of CD103+ DC that limit acute inflammation and inhibit progression of pulmonary fibrosis [21]; as well as repairing airway epithelium and re-epithelialization of airway wounds by facilitating cell migration. MMP family members are also key factors responsible for degradation and tissue remodeling of the extracellular matrix [22]. Several studies have demonstrated the importance of MMPs in tuberculosis as pulmonary damage, mainly granuloma formation, is fundamental to *Mycobacterium tuberculosis* and *Mycobacterium bovis* pathology [23], [24], [25], [26]. A recent study using *M. marinum* infection of zebra fish as a model system, suggested the involvement of mmp-9 in the interaction of bacterial secreted proteins with the host epithelium contributing to early bacteria growth, local expansion and systemic dissemination [27].

Other genes associated with connective tissue development that we found to be differentially modulated in VU lungs were decorin and pentraxin 3. Decorin is a leucine proteoglycan, which is a key regulator of collagen fibrogenesis [28] and TGF-β [29]. It has been localized in epithelioid cells of noncaseating granulomas in TB patients [30]. Pentraxin 3, an acute phase protein produced in lungs following infection and inflammation, correlates with protective immunity against *Aspergillus fumigatus* and *Pseudomonas aeruginosa* in lungs [31] and a specific haplotype frequency was associated with a protective effect against pulmonary tuberculosis in West Africans [32].

Correlates of protection, i.e. markers that are associated with the anamnestic immune response after challenge of vaccinated mice, could be not only useful as biosignatures in clinical trials, but could also be useful to describing protective immunity induced by BCG vaccination. Our study prioritized genes associated with TH17 cell differentiation and effector function in addition to TH1 responses defined by IFN-γ production. However, one cannot rule out that the TH-17 responses found in our study were up-regulated due to BCG persistence in tissues and did not directly correlate with the protective immune response post-challenge. However, our responses occurred post-challenge and this therefore makes it more probable that TH17 responses do contribute to protective immunity. Previously, it has been demonstrated that a population of IL-17-producing cells are induced in lungs by ESAT-6 peptide subunit immunization and their absence compromised TH1 responses and protection [33]. In addition, IL-17 and IL-21 gene expression were up-regulated in lungs from mice vaccinated with BCG or a *M. tuberculosis* Δ*secA2* mutant strain post challenge [11]. Further, Kolibab and co-workers [34] have recently extended these results using sub-unit vaccination based on recombinant viral subunits by demonstrating IL-17 production in the lungs of vaccinated mice post-challenge. Interestingly, we have recently also detected strong TH-17-associated responses associated with protection of cattle after heterologous prime-boost vaccination using BCG and recombinant attenuated viral subunit vaccines by demonstrating increased IL-17 [35], IL-23 and RORγt expression (Aranday-Cortes and Vordermeier, unpublished data) in protected animals. Therefore, we have validated to some degree the findings described in this paper in mice in a target species of vaccination. Taken together, there is therefore a mounting body of evidence that is suggestive of a role of IL-17 in protective immunity. By contrast, the evidence for a protective role of IL-17-associated responses after vaccination with DNA vaccines against tuberculosis appears to be less clear. The study by Lim and co-workers [11] did not demonstrate IL-17 responses in the lungs of mice vaccinated mice with a DNA vaccine after challenge, which is in contrast to their data demonstrating IL-17 responses in mice vaccinated with live mycobacterial vaccines [11]. Furthermore, an earlier study described a negative association of IL-17 production following vaccination and protection following a BCG/DNA heterologous prime-boost protocol [36]. Therefore, these discrepancies between different subunit vaccine types need to be examined in future studies.

In the spleens of vaccinated mice examined in the present study, epx and mpo were differentially expressed after challenge. Both proteins have demonstrated antimycobacterial roles in eosinophils [37], [38]. Proteoglycan-2 or eosinophil granule major basic protein (mbp2) is a specific eosinophil marker [39]. Moreover, mpo, CD177, neutrophilic granule protein (npg), lactotransferrin (ltf) and cathepsin G have been associated with the function of neutrophils [40], [41], [42]. Furthermore, the chitinase 3-like 3 (chi3l3) or ym1, (an alternative activation macrophage marker), is also expressed in neutrophilic granules [43]. It has been demonstrated that eosinophils and neutrophils are attracted to the site of *M. tuberculosis* infection [44] and the role of neutrophils against mycobacterial infection has been evaluated in several studies. For example, neutrophils played a protective role at the early phase of infection with *M. tuberculosis* in one study; where neutrophil depletion significantly compromised host defense [45]. In other studies, immunization with BCG resulted in increased production of the neutrophil chemoattractant IL-8 by alveolar macrophages associated with neutrophilia [46], [47] following challenge. Interestingly, both the up-regulation of genes associated with neutrophil biology and chemoattraction and the differential regulation of TH17-related genes that we observed has been reported by others. Khader et al. [48] also demonstrated reciprocal interaction of neutrophils and Th17 cells via chemokine and cytokine interaction (e.g. IL-17A/F, IL-23, CXCL10, CXCL11 [49], [50]).

In conclusion, our study has prioritized both biomarkers predicting vaccination success before challenge and bio-signatures that are potentially associated with protective immune responses. We are currently assessing these markers in cattle, the target species of vaccines against bovine tuberculosis.

## Supporting Information

Table S1 List of pulmonary biosignature genes found after vaccination.
(0.07 MB XLS)

Table S2 List of biosignature genes found after infection.
(0.02 MB XLS)
